# Agrp-Specific Ablation of Scly Protects against Diet-Induced Obesity and Leptin Resistance

**DOI:** 10.3390/nu11071693

**Published:** 2019-07-23

**Authors:** Daniel J. Torres, Matthew W. Pitts, Ann C. Hashimoto, Marla J. Berry

**Affiliations:** Department of Cell and Molecular Biology, John A. Burns School of Medicine, University of Hawai‘i, Honolulu, HI 96813, USA

**Keywords:** agrp, hypothalamus, leptin, scly, selenium, selenoprotein, thermogenesis, type 2 diabetes

## Abstract

Selenium, an essential trace element known mainly for its antioxidant properties, is critical for proper brain function and regulation of energy metabolism. Whole-body knockout of the selenium recycling enzyme, selenocysteine lyase (Scly), increases susceptibility to metabolic syndrome and diet-induced obesity in mice. Scly knockout mice also have decreased selenoprotein expression levels in the hypothalamus, a key regulator of energy homeostasis. This study investigated the role of selenium in whole-body metabolism regulation using a mouse model with hypothalamic knockout of Scly. Agouti-related peptide (Agrp) promoter-driven Scly knockout resulted in reduced weight gain and adiposity while on a high-fat diet (HFD). Scly-Agrp knockout mice had reduced Agrp expression in the hypothalamus, as measured by Western blot and immunohistochemistry (IHC). IHC also revealed that while control mice developed HFD-induced leptin resistance in the arcuate nucleus, Scly-Agrp knockout mice maintained leptin sensitivity. Brown adipose tissue from Scly-Agrp knockout mice had reduced lipid deposition and increased expression of the thermogenic marker uncoupled protein-1. This study sheds light on the important role of selenium utilization in energy homeostasis, provides new information on the interplay between the central nervous system and whole-body metabolism, and may help identify key targets of interest for therapeutic treatment of metabolic disorders.

## 1. Introduction

The trace element selenium (Se) is a vital micronutrient that promotes redox balance and protects cells from oxidative stress. Selenium is required for the synthesis of selenoproteins, which function in a wide range of biological processes, such as thyroid hormone metabolism [1], fertility [2], and inflammation [3]. While altered Se status and selenoprotein expression have been associated with metabolic disorders such as type 2 diabetes (T2D) and obesity in humans, the mechanisms underlying this relationship are not well understood [4]. In animal models, various metabolic disturbances can be induced by the targeted disruption of different selenoproteins [5,6,7,8], as well as the enzyme selenocysteine lyase (Scly) [9], which is important for intracellular Se utilization [10]. Scly catalyzes the breakdown of the amino acid selenocysteine into alanine and selenide, to be used for de novo selenoprotein synthesis [11,12]. Genetic deletion of Scly in mice results in a striking metabolic phenotype that includes glucose insensitivity and hyperinsulinemia, as well as a greater propensity to develop metabolic syndrome, with males exhibiting more drastic changes [9]. Scly knockout (KO) mice are also more susceptible to high-fat diet (HFD)-induced obesity and related complications [13]. Selenoprotein expression is reduced in several tissues of Scly KO mice, including the hypothalamus, a brain region involved in the regulation of energy metabolism [14]. Growing evidence depicts an association between hypothalamic damage and obesity in both humans and rodents [15].

The importance of Se for proper brain function is well documented [16] and a prominent role for selenoproteins in the hypothalamus is supported by several recent studies. Tagashita et al. demonstrated that broad hypothalamic KO of the selenocysteine-tRNA (Trsp) in mice causes an overweight phenotype [17]. Schriever et al. found that conditional KO of the selenoprotein glutathione peroxidase 4 (GPx4) in agouti-related peptide (Agrp)-positive neurons in the hypothalamus exacerbates diet-induced obesity [18]. Additionally, selenoprotein M (SelM) was found to support hypothalamic leptin signaling [19], which may underlie the obesity phenotype observed in SelM KO mice [7]. Circulating leptin acts upon neurons in the hypothalamus, including inhibiting Agrp neurons, to promote a negative energy balance. Resistance to the anorexigenic actions of leptin is one of the hallmarks of obesity and is strongly associated with T2D [20]. Hypothalamic oxidative stress and endoplasmic reticulum (ER) stress have been implicated as causative factors in leptin resistance development [21], implying that Se may play an important role in maintaining leptin sensitivity. Hypothalamic dysfunction, including impaired leptin signaling, may therefore contribute to the metabolic phenotype of Scly KO mice.

Known as ‘first-order neurons’ in sensing energy homeostasis signals to the brain, Agrp neurons are located within the arcuate nucleus (Arc) and the median eminence (ME) of the hypothalamus, placing them in close proximity to circulating hormones and nutrients in the bloodstream [22]. Interestingly, Agrp neurons have been shown to be particularly susceptible to developing leptin resistance compared to other neuron types [23]. To explore the potential contributions of hypothalamic dysfunction to the metabolic phenotype of whole-body Scly KO mice, we generated a mouse line with Cre-driven Agrp neuron-specific KO of Scly (Scly-Agrp KO mice). Male and female mice were investigated in parallel to characterize any potential sex difference. The results discussed in this report highlight an important role for hypothalamic Scly in mediating HFD-induced weight gain and leptin resistance in mice.

## 2. Materials and Methods

### 2.1. Animals

Agrp^tm1(Cre)Lowl^/J mice with IRES inserted in exon 3 of the Agrp gene were purchased from The Jackson Laboratory (Bar Harbor, ME, USA) [24]. Agrp^tm1(cre)Lowl^/J mice were cross-bred with C57/BL6J mice with loxP sites flanking the Scly gene (Scly^fl/fl^) to generate male and female Scly^fl/fl; Agrp-Cre^ mice (Scly-Agrp KO mice). Littermates with the Scly gene floxed, but lacking the cre-driver (Scly^fl/fl; Agrp-WT^) were used as controls. Comparison of control male mice (Scly^fl/fl; Agrp-WT^) versus mice containing the cre-driver without the Scly gene floxed (Scly^WT; Agrp-Cre^) demonstrated that the cre-driver did not impact HFD-induced weight gain. Although female Scly^WT; Agrp-Cre^ mice gained weight at a faster rate than Scly^fl/fl; Agrp-WT^ mice, this trend was in opposition to the effect observed in experimental (Scly^fl/fl; Agrp-Cre^; Scly-Agrp KO) mice and, thus, did not account for the main findings of the study.

Mice were maintained on a 12-h light/dark cycle and allowed ad libitum food and water access. Sterilized glucose, leptin, and vehicle control (phosphate-buffered saline) were administered via intraperitoneal (i.p.) bolus injection. Mice were either anaesthetized with tribromoethanol via i.p. injection prior to transcardial perfusion or euthanized via CO_2_ asphyxiation prior to fresh tissue collection. All animal experiments and procedures were conducted with the approval of the University of Hawaii’s Institutional Animal Care and Use Committee (IACUC). Animal Care and Use Committee (IACUC) Protocol: APN 09-871-9, approved: 16 August 2018. Institutional Biosafety Committee (IBC) Protocol: IBC #18-10-544-02-4A-1R, approved: 23 October 2018.

### 2.2. Experimental Design

Mice were weaned at 21 days, fed standard lab chow until 4 weeks of age, then switched to a high-fat diet (HFD) chow containing 45% kcal fat and an energy density of 4.7 kcal/g (Research Diets, New Brunswick, NJ, USA; D12451). Body weight was measured at 10:00 every 14 days thereafter. At 16 weeks of age, in vivo metabolic phenotyping was carried out using metabolic chambers to monitor food intake and respiratory metabolism. A glucose tolerance test (GTT) was performed at 18 weeks of age and mice were sacrificed at 24 weeks of age for tissue collection. Mice were fasted overnight for 16 h (18:00 until 10:00 the next day) and injected intraperitoneally with either leptin (1 mg/kg body weight; R & D Systems, Minneapolis, MN, USA; 498-OB) or the vehicle (sterilized phosphate-buffered saline) without leptin 1 h prior to sacrifice. Tissue was either fixed via perfusion for immunohistochemical assays or snap-frozen in liquid nitrogen for Western blot analysis.

### 2.3. Metabolic Chambers

Food intake, activity, and respiratory metabolism were measured in mice using the PanLab Oxylet*Pro*^TM^ System (Harvard Apparatus, Barcelona, Spain) per the manufacturer’s instructions. Mice were placed in individual homecage-like chambers, with fresh bedding, food, and water, and allowed to acclimate for 24 h, followed by 48 h of data collection. Cage air was sampled for 7-mi epochs every 35 min to measure oxygen and carbon dioxide concentrations. Data were collected and analyzed with Panlab METABOLISM software (Vídeňská, Prague, Czech Republic).

### 2.4. Glucose Tolerance Test

Mice were fasted overnight for a total of 16 h (18:00 until 10:00 the next day). At 10:00, blood was drawn via tail vein puncture and baseline glycemia measured using a OneTouch Ultra2 glucometer (Lifescan, Milpitas, CA, USA). Mice were then administered glucose (1 g/kg body weight) via intraperitoneal bolus injection and glycemia measured at 30 min, 1 h, 2 h and 3 h post-injection.

### 2.5. Tissue Collection and Processing

Prior to tissue collection, final body weight and body length were recorded. For immunohistochemical analysis, mice were euthanized with tribromoethanol (1%, 0.1 mL/g body weight), blood collected via cardiac puncture, and perfused transcardially with phosphate buffer, followed by 4% paraformaldehyde (PFA) in phosphate buffer. Brains were collected in 4% PFA for overnight post-fix followed by dehydration via sucrose gradient, then cut into 40 µm floating coronal sections using a cryostat. Floating sections were stored in a cryoprotective solution (50% 0.1 M phosphate buffer, 25% glycerol, 25% ethylene glycol) until analyzed. Brown adipose tissue (BAT) was collected and stored in 4% PFA for several days before being paraffin-embedded and cut into 5 µm sections. For Western blot analysis of fresh-frozen tissue, mice were euthanized with CO_2_, blood collected and inguinal white adipose tissue removed and weighed. Brains were placed in 30% sucrose on ice for 1 min, then the hypothalamus was dissected and snap-frozen in liquid nitrogen.

Frozen hypothalami were pulverized using the CryoGrinder kit (OPS Diagnostics, Lebanon, NJ, USA; CG 08-01). Individual brain parts were placed in the ceramic mortar on dry ice and ground into powder using the ceramic pestle. The powder was added to a tube containing 300 µL CelLytic MT Mammalian Tissue Lysis/Extraction Reagent (Sigma, St. Louis, MO, USA; C-3228) containing a protease/phosphatase inhibitor cocktail (1:100; Cell Signaling, Danvers, MA, USA; 5872) and sonicated with 20 one-second pulses at 5 Hz, separated by one second each, using a Fisher Sonic Dismembrator Model 100 (Fisher Scientific, Hampton, NH, USA). Samples were then centrifuged at 14,000× *g* for 10 min at 4 °C and supernatant was collected and stored at −80 °C for Western blotting.

### 2.6. Gel Electrophoresis and Western Blotting

Hypothalamic lysate samples containing 40 µg of protein were separated on 4%–20% gradient polyacrylamide TGX gels (BIO-RAD, Hercules, CA, USA; 5671094) via electrophoresis and transferred to 0.45 µm pore size Immobilon-FL polyvinylidene difluoride membranes (Millipore, Burlington, MA, USA; IPFL00010). Membranes were incubated in PBS-based blocking buffer (LI-COR Biosciences, Lincoln, NE, USA; P/N 927) for 1 h and then probed with primary antibodies overnight at 4 °C with shaking, followed by washing with PBS containing 0.01% Tween 20 (PBS-T). Blots were incubated with infrared fluorophore-bound secondary antibodies in the dark, washed again with PBS-T, and analyzed using the Odyssey CLx Imaging System (LI-COR Biosciences). After the phosphorylated signal transducer and activator of transcription 3 (pSTAT3) was measured, membranes were stripped (Re-Blot Plus Strong Solution; Millipore, Burlington MA, USA; 2504) and then probed for STAT3 to generate a pSTAT3/STAT3 ratio.

### 2.7. Immunohistochemistry and Histology

For the detection of proteins by 3,3′-diaminobenzidin (DAB), endogenous peroxidases were inactivated with 1% H_2_O_2_ in methanol. Sections were blocked in normal goat serum and incubated overnight at 4 °C in primary antibody. Next, sections were probed with the appropriate biotinylated secondary antibodies, then incubated with an avidin-biotin-peroxidase complex (Elite ABC Kit; Vector Labs, Burlingame, CA, USA; PK-6100). DAB chromogen (DAB Substrate Kit; Vector Labs; H-2200) was used for peroxidase detection of immunoreactivity. Finally, sections were rinsed with PBS, mounted on glass slides, dehydrated by ethanol gradient followed by xylene, and cover-slipped.

To visualize BAT morphology, 5 µm sections were stained with hematoxylin and eosin (H & E). To evaluate UCP1 levels, sections were placed in a 60 °C oven for 30 min, deparaffinized with xylene and ethanol, and incubated in 1% H_2_O_2_ in methanol for 30 min. Antigen retrieval was performed with 0.01 M citric acid (pH 6) and sections were blocked using an avidin/biotin blocking kit (Vector Laboratories, Burlingame, CA, USA; SP-2001), followed by incubation with primary antibody and then biotinylated secondary antibody. DAB staining was performed as described above to visualize protein expression and sections were counterstained with hematoxylin before being dehydrated and cover slipped.

For verification of KO of Scly in Agrp neurons, we performed immunofluorescence on a free-floating section from subjects with Agrp-driven expression of the fluorescent dTomato protein. Sections were blocked with normal goat serum, followed by incubation with goat anti-mouse antibody (Jackson Laboratories, West Grove, PA, USA; 115-007-003). Anti-Scly primary antibody was coupled with Alexa Fluor 488 fluorescent secondary antibody (Abcam, Cambridge, MA, USA; ab150113) (1:2 ratio) for 30 min, non-coupled secondary antibody quenched with mouse serum, and the antibody complex was then added to the sections and incubated overnight at 4 °C with shaking. Representative images were captured on a Leica TSP SP8 HyVolution Confocal Microscope (Leica Biosystems, Buffalo Grove, IL, USA).

### 2.8. Stereology and Data Quantification

Sections at bregma −1.46 mm were used for analysis of the Arc, median eminence (ME), ventromedial hypothalamus (VMH), and dorsomedial hypothalamus (DMH) (illustrated in Supplementary Figure S3). To analyze the paraventricular nucleus (PVN) and periventricular nucleus, sections at bregma −0.94 mm were used. Analysis was performed with Stereo Investigator Software (MBF Bioscience, Williston, VT, USA) on an upright microscope (Axioskop2; Zeiss, Oberkochen, Germany). To quantify Agrp neurons, sections at bregma −2.46 mm were visualized with a 5× objective lens, then the total number of dTomato-positive cells were counted in the arcuate nucleus (Arc) of either the left, right or both hemispheres and the mean was calculated for each subject. To measure the optical density of pSTAT3 and Agrp immunoreactivity, 5× brightfield images were captured and imported into ImageJ software. Images were converted to black-and-white, inverted and the mean value per pixel measured within each region of interest. The ImageJ plugin Cell Counter was used to count pSTAT3-positive cells.

The simple random sampling workflow in Stereo Investigator was used to take up to 10 images of BAT sections stained with H&E. Images were analyzed using the FIJI (ImageJ v2) plugin Adiposoft to count and measure the size of individual lipid droplets, and values were averaged for each subject. To measure UCP1 optical density, images were converted to black-and-white, inverted and the mean pixel value for the entire image was measured. Lipid droplets have the potential to bias data since they comprise a significant percentage of each image and do not express UCP1. To account for this, the mean pixel value for an individual lipid droplet was measured, multiplied by the area of the image filled by lipid deposition, and this value was subtracted from the optical density measured for the entire image to calculate the overall intensity of UCP1 staining.

### 2.9. Antibodies

The following primary antibodies were used: rabbit anti-mouse Agouti-related protein antiserum (1:1000; Alpha Diagnostic International, Inc., San Antonio, TX, USA; AGRP11-S), rabbit anti-phospho-STAT3 (Tyr705) (D3A7) (1:1000; Cell Signaling, Danvers, MA, USA; 9145), rabbit anti-STAT3 (D1A5) (1:1000; Cell Signaling, Danvers, MA, USA; 8768), mouse anti-selenocysteine lyase (32) (1:200; Santa Cruz Biotechnology, Santa Cruz, CA, USA; sc-136394), rabbit anti-β-actin (13E5) (1:5000; Cell Signaling, Danvers, MA, USA; 4970), rabbit anti-uncoupling protein 1 (1:500; Abcam, Cambridge, MA, USA; ab10983).

### 2.10. Statistical Analysis

Statistical tests and sample numbers varied with each assay performed and are indicated in the figure legends. Generally, data sets were analyzed by two-way ANOVA to detect changes caused by genotype and either sex or leptin treatment, and graphed accordingly. Tukey’s multiple comparisons test was used for post-hoc analysis, unless a repeated measures design analysis was performed, in which case, Bonferroni’s multiple comparisons test was used. In cases of immunohistochemical analysis of brain sections, separate two-way ANOVAs were performed for each hypothalamic region to compare genotype and leptin response. Sex-wise statistical comparisons were not performed on DAB-stained brain sections since staining was performed separately. Data were analyzed and plotted using GraphPad Prism version 7 software. All results are represented as mean ± standard error of the mean (SEM). Significance was determined by a *p*-value of <0.05. Sample sizes, ‘*n*’, reported in graphs and figure legends, represent biological replicates. Technical replicates (images from a single section) were sampled from BAT sections, as described in Section 2.8 above, and the mean was calculated for each biological specimen.

## 3. Results

### 3.1. Scly-Agrp KO Are Resistant to HFD-Induced Weight Gain

**Figure 1:**
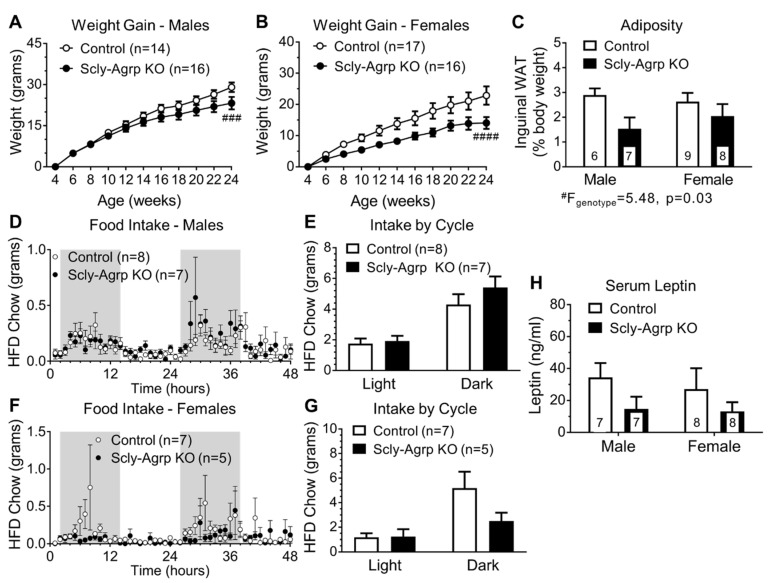
Metabolic characterization of high-fat diet (HFD)-fed Scly-Agrp knockout (KO) vs. control mice. (**A**) Weight gain on a high-fat diet in male Scly-Agrp KO mice vs. controls. Two-way ANOVA: genotype ^###^ F(_1,308_) = 14.24, *p* = 0.0002; (**B**) Weight gain in females. Two-way ANOVA: genotype ^####^ F(_1,341_) = 48.88, *p* < 0.0001; (**C**) Inguinal fat deposits, expressed as percent body weight, by sex and genotype. Two-way ANOVA: genotype ^#^ F(_1,26_) = 5.48, *p* = 0.03; (**D**) Food consumption over a 48-h period in male mice. Gray shading indicates dark cycle; (**E**) Comparison of total intake in males by cycle: Two-way ANOVA: genotype F(_1,26_) = 1.349, *p* = 0.26; (**F**) Food consumption in female mice; (**G**) Comparison of total intake in females by cycle. Two-way ANOVA: genotype F(_1,20_) = 2.118, *p* = 0.16, interaction F(_1,20_) = 2.303, *p* = 0.14; (**H**) Fasting serum leptin levels in male and female mice by genotype. Two-way ANOVA: genotype F(_1,26_) = 3.188, *p* = 0.086. All data are represented as mean ± standard error of the mean. Group numbers are indicated in each graph.

Knockout of Scly was verified via immunofluorescence (Supplementary Materials Figure S1). Scly-Agrp KO mice and littermate controls were placed on HFD beginning at 4 weeks of age and metabolically evaluated thereafter. Surprisingly, both male and female Scly-Agrp KO mice gained less weight while on HFD than controls (Figure 1A,B), and had reduced inguinal fat deposits (Figure 1C). A comparison of the total body weight of both sexes of control and Scly-Agrp KO mice on HFD over time gave the same result (Supplementary Materials Figure S2C,D). Body length and the ratio of body weight to length were also reduced in Scly-Agrp KO mice (Supplementary Materials Figure S2E,F). Despite gaining less weight, Scly-Agrp KO mice did not exhibit any change in food consumption, although there was a downward trend in females during the dark phase (Figure 1D–G). While there were no significant changes in glucose tolerance (Supplementary Materials Figure S2G–I), fasting serum leptin levels trended towards a decrease in Scly-Agrp KO mice, possibly reflecting a change in leptin sensitivity (Figure 1H). Overall, metabolic characterization of Scly-Agrp KO mice revealed an anorexigenic phenotype that is in stark contrast to whole-body Scly KO mice.

### 3.2. Scly-Agrp KO Mice Do Not Exhibit HFD-Induced Leptin Resistance in the Arcuate Nucleus.

**Figure 2:**
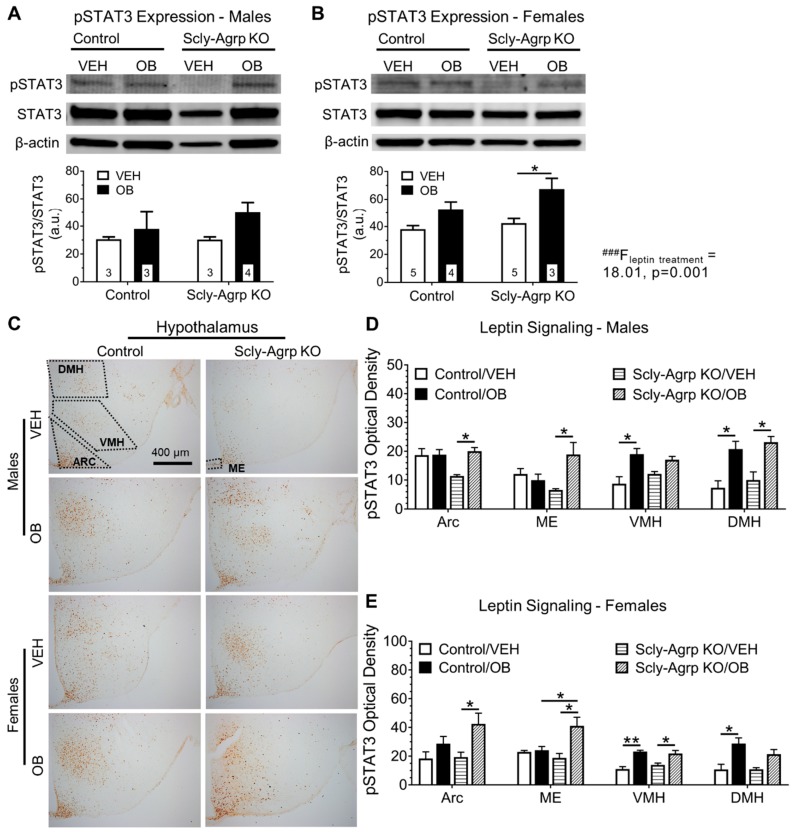
Leptin challenge-induced signaling in the hypothalamus of HFD-fed Scly-Agrp KO vs. control mice. (**A**) Western blot analysis of phosphorylated STAT3 (pSTAT3) levels in hypothalamic protein lysates from male mice, following intraperitoneal (i.p.) injection of leptin (Ob, 1 mg/kg body weight) or vehicle control (VEH, phosphate-buffered saline). Two-way ANOVA: leptin treatment F(_1,9_) = 3.26, *p* = 0.1; (**B**) pSTAT3 levels in female hypothalamic protein lysates. Two-way ANOVA: leptin treatment ^###^ F(_1,13_) = 18.01, *p* = 0.001; (**C**) Sample images of hypothalamic sections stained for pSTAT3 at 10× magnification; (**D**) Optical density of pSTAT3 measured in male mice in the arcuate nucleus (Arc): Two-way ANOVA: leptin F(_1,9_) = 5.889, *p* = 0.038, interaction F(_1,9_) = 5.537 *p* = 0.043, median eminence (ME): interaction F(_1,9_) = 8.409, *p* = 0.018, ventromedial hypothalamus (VMH): leptin F(_1,10_) = 15.02, *p* = 0.003, and dorsomedial hypothalamus (DMH): leptin F(_1,8_) = 27.9, *p* = 0.0007; (**E**) pSTAT3 optical density in female mouse; Arc: Two-way ANOVA: leptin F(_1,10_) = 8.777, *p* = 0.014, ME: leptin F(_1,8_) = 10.35, *p* = 0.012, interaction F(_1,8_) = 8.4 *p* = 0.02, VMH: leptin F(_1,10_) = 36.54, *p* = 0.0001, and DMH: leptin F(_1,8_) = 19.96, *p* = 0.002. All data are represented as mean ± standard error of the mean. Group numbers are indicated in graphs. Group numbers for (**D**,**E**) ranged from 3–5. Tukey’s multiple comparisons test: * *p* < 0.05, ** *p* < 0.01.

High-fat diet consumption promotes leptin resistance in rodents [25,26]. Western blot analysis of whole-hypothalamus lysates from leptin-injected (1 mg/kg body weight) control mice fed a HFD did not reveal a significant change in phosphorylation of the leptin signaling protein signal transducer and activator of transcription (pSTAT3), indicating that the mice had developed leptin resistance. Scly-Agrp KO mice fed a HFD, however, exhibited an increase in pSTAT3 in response to leptin, demonstrating that leptin sensitivity was maintained in this group (Figure 2A,B). Immunohistochemical interrogation of hypothalamic regions (see Supplementary Materials Figure S3 for anatomical delineation) revealed that control mice developed leptin resistance in the arcuate nucleus (Arc) and median eminence (ME), which contain the Agrp neuron cell bodies (Figure 2C–E). The dorsomedial hypothalamus (DMH) and ventromedial hypothalamus (VMH), both leptin-responsive regions that do not contain Agrp neurons, maintained leptin sensitivity. Scly-Agrp KO mice, on the other hand, exhibited a robust increase in pSTAT3 in all regions, including the Arc, in response to leptin (Figure 2C–E). This same effect was observed whether measuring pSTAT3 optical density or counting pSTAT3-positive cells (Supplementary Materials Figure S4). These results indicate that while HFD induced Arc- and ME-specific leptin resistance in control mice, Scly-Agrp KO mice were protected from developing leptin resistance.

### 3.3. Scly-Agrp KO Mice Have Less Agrp Neurons and Reduced Hypothalamic Agrp Immunoreactivity Compared to Controls

**Figure 3:**
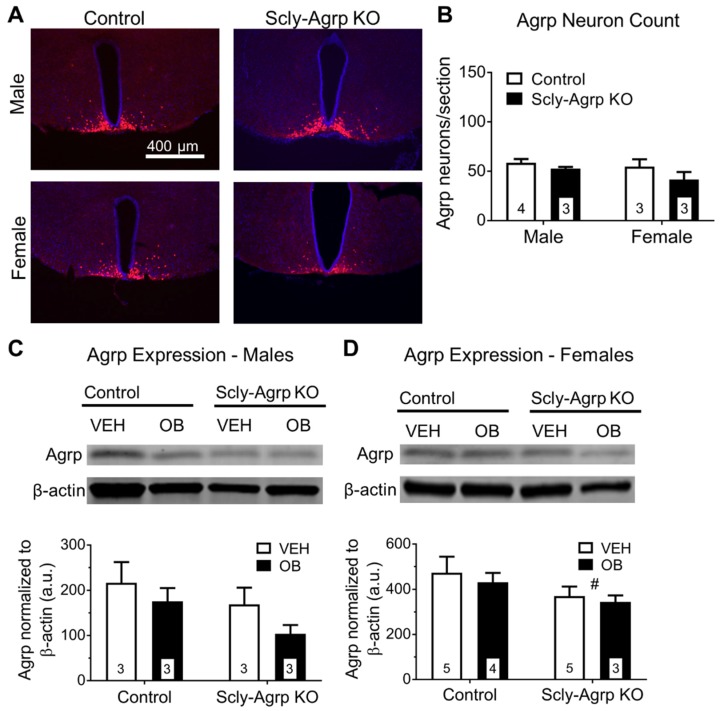
Agrp neuron count and hypothalamic Agrp protein expression in HFD-fed Scly-Agrp KO vs control mice. (**A**) Sample images of hypothalamic sections with dTomato-positive (red) Agrp neurons, in Scly-Agrp KO mice and Scly^WT;Agrp-Cre^ mice used as controls, counter-stained with 4′6-diamidino-2-phenyllindole (DAPI; blue), at 10× magnification; (**B**) Agrp neuron counts trended towards a reduction in male and female Scly-Agrp KO mice. Two-way ANOVA: genotype ^#^ F(_1,9_) = 3.016, *p* = 0.12; (**C**) Agrp expression in hypothalamic protein lysates from males injected with vehicle (VEH) or leptin (OB), measured via Western blot and expressed as arbitrary units (a.u.). Two-way ANOVA: genotype F(_1,8_) = 4.73, *p* = 0.12, leptin treatment F(_1,8_) = 2.41, *p* = 0.16; (**D**) Agrp expression in females. Two-way ANOVA: genotype ^#^ F(_1,8_) = 3.03, *p* = 0.049. All data are represented as mean ± standard error of the mean. Group numbers are indicated in each graph. # denotes a significant genotype effect.

The under-weight phenotype displayed by Scly-Agrp KO mice while on HFD suggests that the overall influence of Agrp neurons may be reduced. The Scly-Agrp KO mice also have Agrp-Cre-driven expression of a dTomato reporter gene. The number of dTomato-positive cells trended towards a reduction in hypothalamic sections from Scly-Agrp KO mice, suggesting the presence of fewer Agrp neurons (Figure 3A,B). Total hypothalamic Agrp expression measured via Western blot was significantly reduced in female Scly-Agrp KO mice (Figure 3C,D) while trending downward in males. Thus, Scly-Agrp KO mice appear to have less Agrp-ergic activity, which likely drives the metabolic phenotype observed.

**Figure 4:**
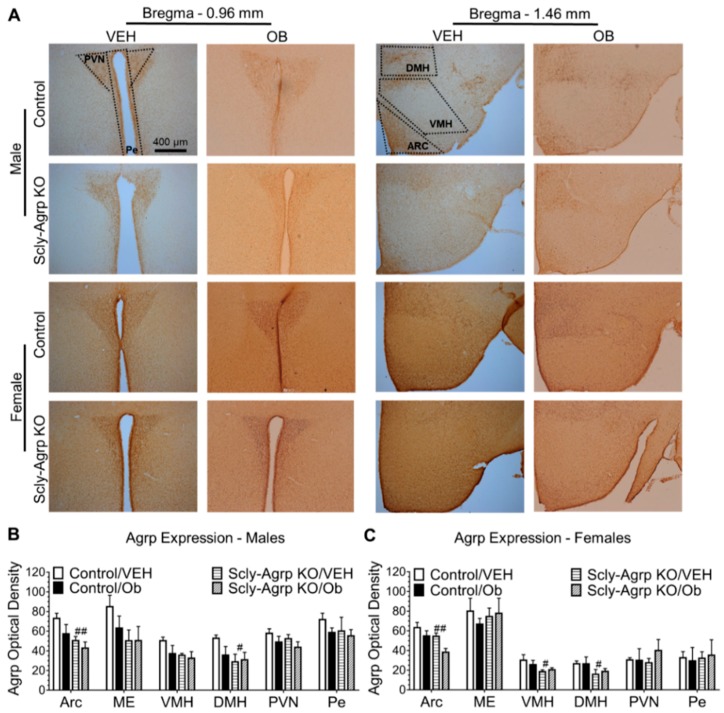
Agrp immunoreactivity in hypothalamus of HFD-fed Scly-Agrp KO vs control mice. (**A**) Sample images of hypothalamic sections showing Agrp immunoreactivity at 10× magnification; (**B**) Agrp expression in hypothalamic sections from male mice, injected with either vehicle (VEH) or leptin (OB), analyzed via two-way ANOVA in the arcuate nucleus (Arc): ^##^ genotype F_(1,10)_ = 10.54, *p* = 0.009, leptin treatment F(_1,10_) = 4.11, *p* = 0.07, median eminence (ME), ventromedial hypothalamus (VMH), dorsomedial hypothalamus (DMH): ^#^ genotype F_(1,9)_ = 1.541, *p* = 0.043, paraventricular nucleus (PVN): leptin treatment F(_1,10_) = 4.04, *p* = 0.072, and periventricular nucleus (Pe); (**C**) Agrp expression in female mice by hypothalamic region: Two-way ANOVA: Arc: ^##^ genotype F_(1,10)_ = 11.9, *p* = 0.006, leptin treatment F_(1,10)_ = 11.36, *p* = 0.007, VMH: ^#^ genotype F_(1,10)_ = 8.87, *p* = 0.014, DMH: ^#^ genotype F_(1,10)_ = 5.38, *p* = 0.04. All data are represented as mean ± standard error of the mean. Group numbers ranged from 3–5. # denotes a significant genotype effect within a particular brain region.

Agrp neurons project to multiple regions of the hypothalamus, including other cells within the Arc, to affect the energy balance [22]. Therefore, we measured Agrp expression in these areas to determine whether Agrp innervation was reduced within a particular neurological circuit (see Supplementary Materials Figure S3 for anatomical delineation). Agrp immunoreactivity was found to be reduced in the Arc and DMH of both male and female Scly-Agrp KO mice (Figure 4A–C). This strongly implies that Scly-Agrp KO mice have reduced Agrp circuitry to these specific hypothalamic regions, which may mediate the underweight phenotype of Scly-Agrp KO mice. 

Leptin regulates Agrp production in the mouse hypothalamus [27]. Interestingly, two-way ANOVA revealed a significant effect of leptin treatment on Agrp expression in the Arc of female mice (Figure 4C). Post-hoc analysis revealed a significant reduction in Agrp expression in response to leptin in the Arc of female Scly-Agrp KO mice, but not in control mice. Leptin had a similar effect in male mice, although the results were not statistically significant (Figure 4B). Considering that Scly-Agrp KO mice maintained leptin sensitivity on HFD while control mice developed leptin resistance (Figure 2), this result provides further insight into a potential mechanism involving leptin regulation of Agrp activity in Scly-Agrp KO mice.

### 3.4. Brown Adipose Tissue Morphology Is Altered in Scly-Agrp KO Mice.

**Figure 5:**
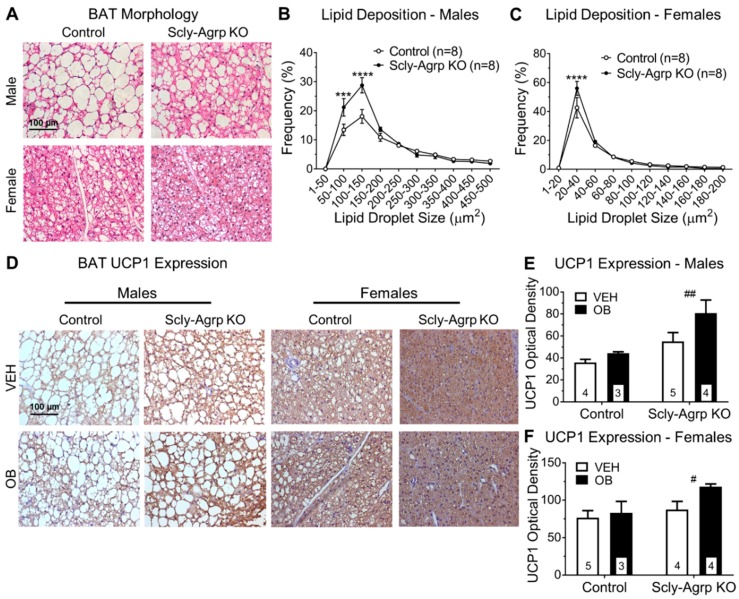
Brown adipose tissue lipid deposition and thermogenesis in HFD-fed Scly-Agrp KO vs control mice. (**A**) Sample images of brown adipose tissue (BAT) sections stained with hematoxylin and eosin, showing adipocytes and lipid droplets at 40× magnification; (**B**) frequency distribution of lipid droplet size in BAT sections from male mice. Two-way ANOVA with repeated measures: genotype F_(1,14)_ = 8.079, *p* = 0.013, interaction F_(9,126)_ = 5.726, *p* < 0.0001, Bonferroni’s multiple comparisons test: *** *p* < 0.001, **** *p* < 0.0001; (**C**) BAT lipid droplet size in female mice. Two-way ANOVA with repeated measures: interaction F_(9,126)_ = 2.418, *p* = 0.014, Bonferroni’s multiple comparisons test: **** *p* < 0.0001. Treatment with either vehicle (VEH) or leptin (OB) did not affect lipid droplet size; (**D**) sample images of uncoupling protein-1 (UCP1) immunoreactivity in BAT sections counter-stained with hematoxylin; (**E**) UCP1 expression in male BAT. Two-way ANOVA: ^##^ genotype F_(1,12)_ = 11.6, *p* = 0.005, leptin treatment F_(1,12)_ = 4.35, *p* = 0.06; interaction F_(1,12)_ = 1.407, *p* = 0.26 (**F**) UCP1 in female BAT. Two-way ANOVA: ^#^ genotype F_(1,12)_ = 5.0, *p* = 0.04, leptin treatment F_(1,12)_ = 3.27, *p* = 0.09, interaction F_(1,12)_ = 1.35, *p* = 0.3. All data are represented as mean ± standard error of the mean. Group numbers are indicated in each graph. # denotes a significant genotype effect.

Although Agrp neurons are primarily known to influence energy metabolism by promoting feeding behavior, there is growing evidence for a prominent role of Agrp neurons in regulating brown adipose tissue (BAT) thermogenesis [28,29,30]. Since the Scly-Agrp KO mice did not exhibit any changes in food intake, we investigated BAT for changes in morphology and thermogenesis. Analysis of H&E-stained BAT sections revealed that lipid droplets in HFD-fed Scly-Agrp KO mice were significantly smaller than in HFD-fed controls, suggesting elevated thermogenesis (Figure 5A–C). While this effect was more pronounced in the male mice, the female mice generally had smaller lipid droplets to begin with, which is consistent with past literature [31]. Overall fat deposition, measured as the fraction of space occupied by lipid mass, was also reduced in Scly-Agrp KO mice (Supplementary Materials Figure S5). 

Mitochondrial uncoupling protein 1 (UCP1) activates BAT thermogenesis, which promotes energy expenditure and fat loss [32]. BAT sections from Scly-Agrp KO mice had significantly greater amounts of UCP1 immunoreactivity than BAT sections from control mice, suggesting elevated thermogenic activity in Scly-Agrp KO mice (Figure 5D,E). These data implicate BAT thermogenesis as a contributing factor in the anorexigenic effect of Agrp neuron-specific KO of Scly.

## 4. Discussion

This study was initiated with the hypothesis that knocking out Scly in Agrp neurons would re-capitulate the obesogenic phenotype of whole-body Scly KO mice [13]. Unexpectedly, Scly-Agrp KO mice gained less weight and adiposity than controls while on HFD (Figure 1), demonstrating the complexity of the role of Scly in hypothalamic function. Previous work on whole-body Scly KO mice revealed decreased expression of multiple selenoproteins in the hypothalamus, including glutathione peroxidase 1 (GPx1), which breaks down hydroperoxides, thus reducing oxidative stress, as well as SelM and selenoprotein S (SelS), both of which mitigate ER stress [14]. The role of ER stress in causing leptin resistance has been established in previous studies using HFD-fed rodents [25,26] and, recently, SelM has been implicated as a mediator of leptin signaling [19]. However, knocking out Scly in Agrp neurons did not cause leptin resistance in our study, and instead conferred protection from Arc- and ME-specific leptin resistance (Figure 2). Previous work by Diano et al., however, demonstrated the capacity of reactive oxygen species (ROS) to suppress Agrp activity [33]. In this study, intracerebroventricular injection of the ROS scavenger honokiol increased Agrp neuron activity, while ROS induction via administration of GW99662, antagonist of peroxisome proliferator-activated receptor-γ (PPAR-γ), suppressed the firing rate of Agrp neurons. Thus, it is possible that the loss of selenoproteins that limit ROS levels, such as GPx1, permitted an overall reduction in Agrp neuron activity in Scly-Agrp KO mice that outweighed any reduction in Agrp neuron leptin sensitivity.

Scly-Agrp KO mice may have had fewer Agrp neurons than controls and less Agrp expression throughout the hypothalamus (Figure 3 and Figure 4), which could contribute to the overall anorexigenic phenotype. One explanation is that oxidative stress induced by the loss of Scly led to the progressive degeneration of some Agrp neurons. Under-weight phenotypes similar to what we observed in the Scly-Agrp KO mice have been reported in other studies in which progressive degeneration of Agrp neurons was induced. For example, while studying a mouse model in which Agrp neurons were progressively ablated by deleting the mitochondrial transcription factor A (Tfam), Xu et al. observed that the mutant mice had reduced body weight and adiposity [34]. Subsequent investigation of the same mouse model by Pierce and Xu revealed that, in response to Agrp neuron degeneration, the hypothalamus generated new cells, some of which became Agrp neurons [35]. Interestingly, this hypothalamic de novo neurogenesis described by the authors gave rise to leptin-responsive cells. It is, therefore, possible that a similar mechanism involving the generation of new leptin-sensitive neurons to replace degenerating Agrp neurons may have contributed to the apparent resistance of Scly-Agrp KO mice to Arc- and ME-specific leptin resistance.

Although Scly-Agrp KO mice gained less weight and had reduced adiposity compared to control mice, no changes in food intake were observed (Figure 1). This is consistent with the feeding behavior of whole-body Scly KO mice which, although they display hyperphagia when placed on a Se-deficient diet [9], do not consume more food than controls while on HFD [13]. Agrp neurons are typically described as promoting feeding behavior as part of the melanocortin system by inhibiting melanocortin-4 receptor (MC4R)-positive neurons in the PVN of the hypothalamus to suppress anorexigenic hormone signals targeting the pituitary gland. However, in addition to observing no change in food intake in Scly-Agrp KO mice, we also found that Agrp immunoreactivity in the PVN was similar between controls and KO mice. Although an effect on the melanocortin system cannot be ruled out, Agrp neurons project to other hypothalamic regions that may also contribute to the anorexigenic phenotype observed. Immunohistochemical analysis showed decreased Agrp expression in one such area, the DMH (Figure 4), which is a known regulator of BAT thermogenesis [36]. Glutamatergic neurons in the DMH project onto sympathetic premotor neurons in the rostral raphe pallidus (rRPa) which subsequently promote BAT thermogenesis via brain stem circuitry [37,38,39]. Agrp neurons provide tonic inhibitory input to the DMH, which limits sympathetic nerve activity [40]. Disinhibiting DMH glutamatergic neurons, as would be the case with decreased input from Agrp neurons, has been shown to stimulate BAT thermogenesis [41]. Interestingly, Scly-Agrp KO mouse BAT sections had smaller amounts of lipid deposition and increased UCP1 expression, suggesting elevated levels of thermogenesis (Figure 5). Together these data implicate enhanced BAT thermogenesis, possibly involving the DMH-sympathetic pathway, as an underlying cause of the resistance to HFD-induced weight gain exhibited by Scly-Agrp KO mice.

The results discussed in this report elucidate a previously undescribed role of Scly in regulating body composition via the hypothalamus. We have shown that loss of Scly within a neuronal sub-population that makes up a small fraction of the hypothalamus [22] produces a phenotype in mice distinguished by reduced weight gain while on HFD and resistance to the development of leptin insensitivity. Moreover, our data reveal yet another way that Scly can influence energy homeostasis: via Agrp neuron-mediated BAT activation. Overall, these findings provide novel insights into the importance of Se utilization in central nervous system-directed energy metabolism.

## Supplementary Materials

The following are available online at https://www.mdpi.com/2072-6643/11/7/1693/s1, Figure S1: Verification of Scly KO in Agrp neurons. Immunofluorescent labeling of Scly in sections of arcuate nucleus in control and Scly-Agrp KO mice taken at 40× and counter-stained with 4′6-diamidino-2-phenyllindole (DAPI; blue). Agrp neurons are positive for dTomato fluorescent protein expression (red) and are labeled with orange arrows. Agrp neurons are positive for Scly (green) in sections from control mice, whereas Agrp neurons in sections from Scly-Agrp KO mice are not positive for Scly, Figure S2: Comparison of weight gain in Scly^fl/fl; Agrp-WT^ vs Scly^WT; Agrp-Cre^ mice and comparison of body length, total body weight and glucose tolerance in Scly-Agrp KO vs control mice. (**a**) Change in total body weight in male Scly-Agrp KO mice vs controls. Two-way ANOVA: genotype F_(1,308)_ = 50.24, *p* < 0.0001; (**b**) Body weight change in females. Two-way ANOVA: genotype F_(1,341)_ = 40.44, *p* < 0.0001; (**c**) Scly^fl/fl; Agrp-WT^ vs Scly^WT; Agrp-Cre^ weight gain in males. Two-way ANOVA: genotype F_(1,253)_ = 3.866, *p* = 0.05; (**d**) and females: genotype F_(1,307)_ = 5.241, *p* = 0.02; (**e**) Body lengths of Scly-Agrp KO vs control mice. Two-way ANOVA: genotype F_(1,53)_ = 8.093, *p* = 0.006; (**f**) Body weight/body length. Two-way ANOVA: genotype F_(1,48)_ = 9.635, *p* = 0.003; (**g**) Time-course of blood glycemia following i.p. bolus injection of glucose (1g/kg body weight) in males; (**h**) and females; (**i**) Comparison of area under the curve representing cumulative glycemia scores over a 3-h period post-injection. Two-way ANOVA: genotype F_(1,45)_ = 0.1589, *p* = 0.69, sex F_(1,45)_ = 8.107, *p* = 0.007. All data are represented as mean ± standard error of the mean. Group numbers are indicated in each graph. Tukey’s multiple comparisons test: * *p* < 0.05. Figure S3: Representative images of hypothalamic sections stained for Agrp, at 5X magnification, to show brain regions. (**a**) Bregma −0.94mm hypothalamus containing paraventricular nucleus (PVN) and periventricular nucleus (Pe); (**b**) Bregma −1.46mm containing arcuate nucleus (Arc), median eminence (ME), ventromedial hypothalamus (VMH), and dorsomedial hypothalamus (DMH), Figure S4: Number of phosphorylated STAT3 (pSTAT3)-positive cells counted in response to leptin (Ob, 1mg/kg body weight) or vehicle (VEH, phosphate-buffered saline) injection in Scly-Agrp KO vs control mice. (**a**) Cell counts of pSTAT3-positive cells measured in male mouse arcuate nucleus (Arc): Two-way ANOVA: leptin F(1,9) = 11.47, *p* = 0.008, genotype F(1,9) = 7.06 *p* = 0.026, median eminence (ME): genotype F(1,9) = 4.421 *p* = 0.065, ventromedial hypothalamus (VMH): leptin F(1,10) = 17.29, *p* = 0.002, and dorsomedial hypothalamus (DMH): leptin F(1,9) = 9.126, *p* = 0.015; (**b**) pSTAT3-cells in female mouse Arc: Two-way ANOVA: leptin F(1,10) = 7.318, *p* = 0.02, ME: leptin F(1,8) = 12.3, *p* = 0.008, VMH: leptin F(1,10) = 31.81, *p* = 0.0002, genotype F(1,10) = 16.42, *p* = 0.002, interaction F(1,10) = 5.543, p = 0.04 and DMH: leptin F(1,8) = 29.8, *p* = 0.0006, genotype F(1,8) = 6.951 *p* = 0.03. All data are represented as mean ± standard error of the mean. Group numbers are indicated in each graph. Tukey’s multiple comparisons test: * *p* < 0.05, ** *p* < 0.01, Figure S5: Brown adipose tissue (BAT) lipid fraction represented as the percent of surface area of the section occupied by lipid droplet. Two-way ANOVA: genotype F(1,28) = 6.474, *p* = 0.017, sex F(1,28) = 4.894, *p* = 0.035. All data are represented as mean ± standard error of the mean. Group numbers are indicated in each graph.
