# Supplementary material for: Agrp-Specific Ablation of Scly Protects against Diet-Induced Obesity and Leptin Resistance
Source: Nutrients. 2019 Jul 23;11(7):1693. doi: 10.3390/nu11071693 (PMC6682868; doi:10.3390/nu11071693)
Supplement: Supplementary file 1 [file nutrients-11-01693-s001.pdf]

nutrients

MDPI

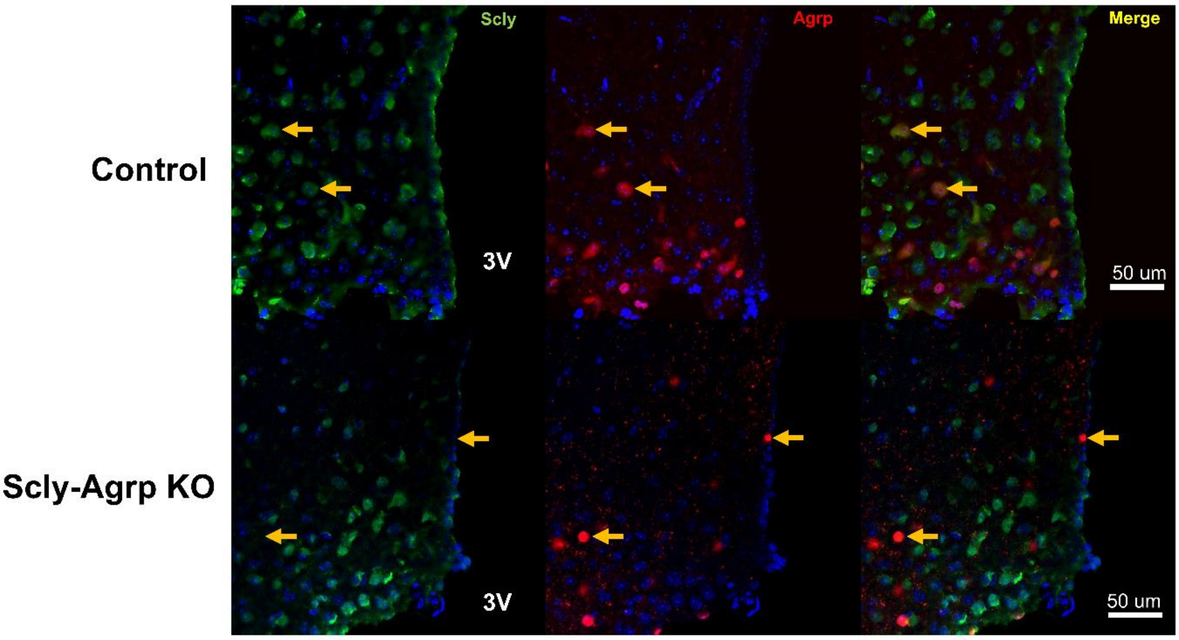


**Supplementary Figure 1.** Verification of Scly KO in Agrp neurons. Immunofluorescent labeling of Scly in sections of arcuate nucleus in control and Scly-Agrp KO mice taken at 40X and counter-stained with 4′6-diamidino-2-phenyllindole (DAPI; blue). Agrp neurons are positive for dTomato fluorescent protein expression (red) and are labeled with orange arrows. Agrp neurons are positive for Scly (green) in sections from control mice, whereas Agrp neurons in sections from Scly-Agrp KO mice are not positive for Scly.

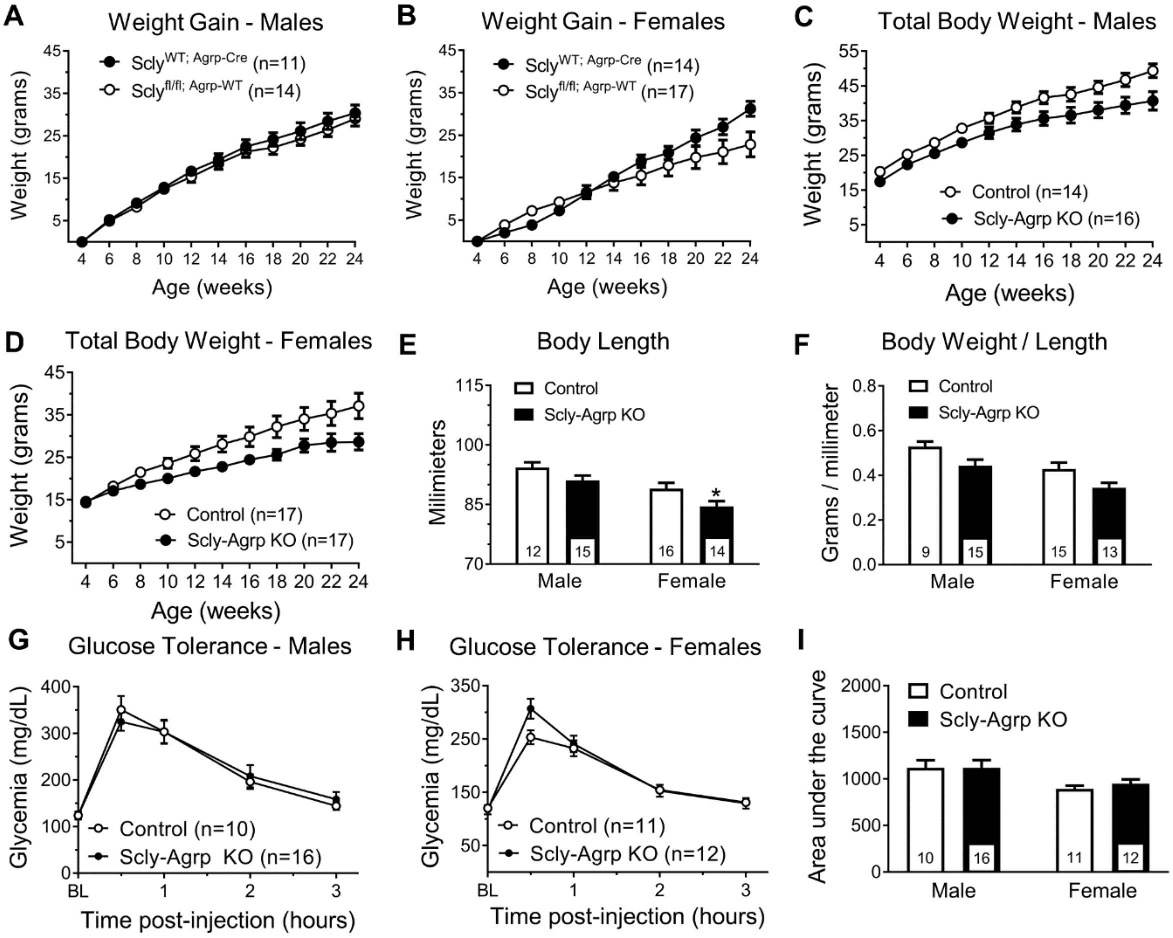

**Supplementary Figure 2.** Comparison of weight gain in Scly$^{fl/fl;\ Agrp\text{-}WT}$ vs Scly$^{WT;\ Agrp\text{-}Cre}$ mice and comparison of body length, total body weight and glucose tolerance in Scly-Agrp KO vs control mice. (**a**) Change in total body weight in male Scly-Agrp KO mice vs controls. Two-way ANOVA: genotype $F_{(1,308)} = 50.24$, $p < 0.0001$; (**b**) Body weight change in females. Two-way ANOVA: genotype $F_{(1,341)} = 40.44$, $p < 0.0001$; (**c**) Scly$^{fl/fl;\ Agrp\text{-}WT}$ vs Scly$^{WT;\ Agrp\text{-}Cre}$ weight gain in males. Two-way ANOVA: genotype $F_{(1,253)} = 3.866$, $p = 0.05$; (**d**) and females: genotype $F_{(1,307)} = 5.241$, $p = 0.02$; (**e**) Body lengths of Scly-Agrp KO vs control mice. Two-way ANOVA: genotype $F_{(1,53)} = 8.093$, $p = 0.006$; (**f**) Body weight/body length. Two-way ANOVA: genotype $F_{(1,48)} = 9.635$, $p = 0.003$; (**g**) Time-course of blood glycemia following i.p. bolus injection of glucose (1g / kg body weight) in males; (**h**) and females; (**i**) Comparison of area under the curve representing cumulative glycemia scores over a 3-hr period post-injection. Two-way ANOVA: genotype $F_{(1,45)} = 0.1589$, $p = 0.69$, sex $F_{(1,45)} = 8.107$, $p = 0.007$. All data are represented as mean ± standard error of the mean. Group numbers are indicated in each graph. Tukey's multiple comparisons test: $*p < 0.05$.

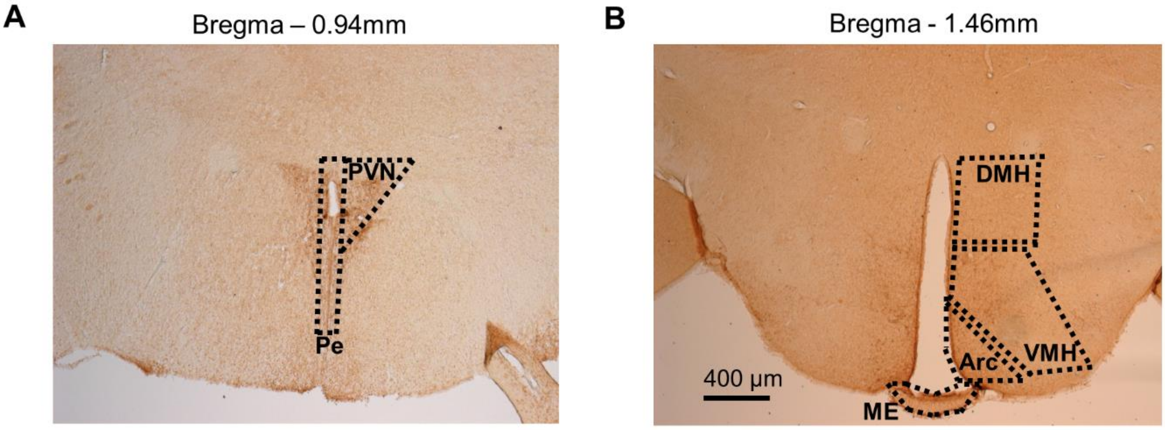


**Supplementary Figure 3.** Representative images of hypothalamic sections stained for Agrp, at 5X magnification, to show brain regions. (**a**) Bregma – 0.94mm hypothalamus containing paraventricular nucleus (PVN) and periventricular nucleus (Pe); (**b**) Bregma – 1.46mm containing arcuate nucleus (Arc), median eminence (ME), ventromedial hypothalamus (VMH), and dorsomedial hypothalamus (DMH).

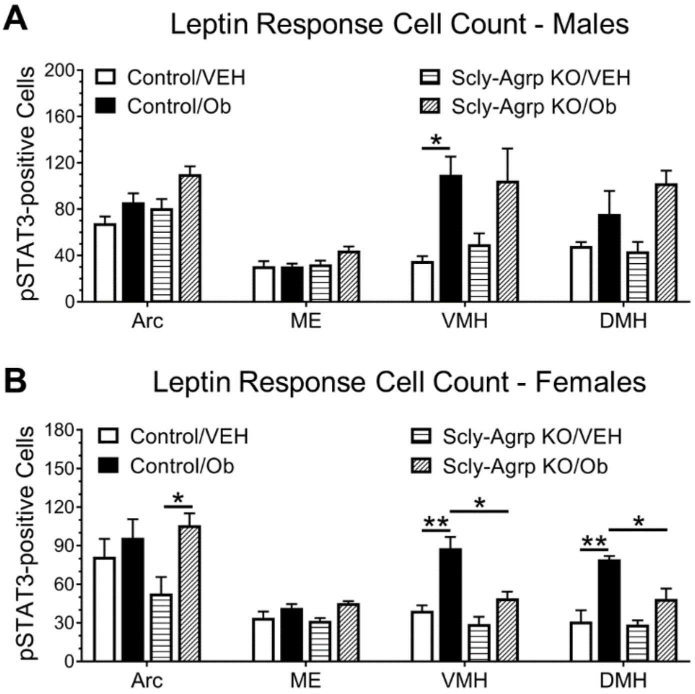

**Supplementary Figure 4.** Number of phosphorylated STAT3 (pSTAT3)-positive cells counted in response to leptin (Ob, 1mg/kg body weight) or vehicle (VEH, phosphate-buffered saline) injection in Scly-Agrp KO vs control mice. (**a**) Cell counts of pSTAT3-positive cells measured in male mouse arcuate nucleus (Arc): Two-way ANOVA: leptin $F_{(1,9)} = 11.47$, $p = 0.008$, genotype $F_{(1,9)} = 7.06$ $p = 0.026$, median eminence (ME): genotype $F_{(1,9)} = 4.421$ $p = 0.065$, ventromedial hypothalamus (VMH): leptin $F_{(1,10)} = 17.29$, $p = 0.002$, and dorsomedial hypothalamus (DMH): leptin $F_{(1,9)} = 9.126$, $p = 0.015$; (**b**) pSTAT3-cells in female mouse Arc: Two-way ANOVA: leptin $F_{(1,10)} = 7.318$, $p = 0.02$, ME: leptin $F_{(1,8)} = 12.3$, $p = 0.008$, VMH: leptin $F_{(1,10)} = 31.81$, $p = 0.0002$, genotype $F_{(1,10)} = 16.42$, $p = 0.002$, interaction $F_{(1,10)} = 5.543$, $p = 0.04$ and DMH: leptin $F_{(1,8)} = 29.8$, $p = 0.0006$, genotype $F_{(1,8)} = 6.951$ $p = 0.03$. All data are represented as mean ± standard error of the mean. Group numbers are indicated in each graph. Tukey's multiple comparisons test: *$p < 0.05$, **$p < 0.01$.

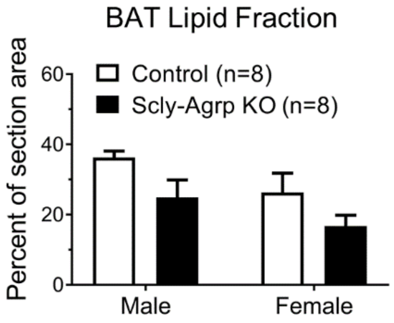


**Supplementary Figure 5.** Brown adipose tissue (BAT) lipid fraction represented as the percent of surface area of the section occupied by lipid droplet. Significant results via Two-way ANOVA: genotype $F_{(1,28)} = 6.474$, $p = 0.017$, sex $F_{(1,28)} = 4.894$, $p = 0.035$. All data are represented as mean ± standard error of the mean. Group numbers are indicated in each graph.
